# Nutrient storage and release in uninfected cells of soybean nodules support symbiotic nitrogen fixation in infected cells

**DOI:** 10.1007/s42994-025-00247-y

**Published:** 2025-09-16

**Authors:** Qian Liu, Qian Dong, Zhi-Chang Chen

**Affiliations:** 1 College of Resources and Environment, Fujian Agriculture and Forestry University, Fuzhou, 350002 China; 2 State Key Laboratory of Soil and Sustainable Agriculture, Institute of Soil Science, Chinese Academy of Sciences, Nanjing, 211135 China

**Keywords:** Callose, Infected cell, Nodule, Plasmodesmata, Uninfected cell

## Abstract

Symbiotic nitrogen fixation (SNF) between legumes and rhizobia contributes to sustainable agriculture. In root nodules, infected cells (ICs) are the primary sites of rhizobial colonization and nitrogen fixation. However, the function of the neighboring uninfected cells (UCs) has received little attention and is poorly understood. In this study, we employed a symplastic tracing approach to elucidate the role of UCs in nutrient storage and transport within root nodules. We uncovered an extensive network of plasmodesmata connecting ICs and UCs, while direct IC–IC connections were absent. By artificially inducing callose deposition at plasmodesmata, we demonstrate that plasmodesmata permeability between ICs and UCs regulates nutrient import into ICs, thereby influencing nutrient homeostasis and the SNF ability of nodules. Furthermore, high nitrogen levels triggered callose deposition at plasmodesmata, restricting nutrient transport, which may represent one mechanism by which excessive nitrogen inhibits SNF. These findings provide insights into the regulatory mechanisms of SNF and underscore the crucial role of UCs in optimizing nitrogen fixation efficiency.

**Supplementary Information:**

The online version contains supplementary material available at 10.1007/s42994-025-00247-y.

## Introduction

Nitrogen (N) is the most abundant element in the atmosphere. However, most plants cannot directly utilize atmospheric nitrogen (N_2_) as a N source for growth. By contrast, legumes possess a unique symbiotic nitrogen fixation (SNF) system that enables them to convert N_2_ into an organic form. This conversion relies on a symbiotic relationship between legumes and rhizobia (Wang et al. 2003; Yu and Zhu 2024; Xu et al. 2017). The SNF system fixes approximately 40 million tons of N_2_ annually, accounting for about 65% of the total biological N fixation in the agricultural system (Herridge et al. 2008). Thus, SNF supplies N to legumes and reduces agricultural reliance on chemical fertilizers, playing a substantial role in sustainable agriculture.

In the symbiotic system, nutrient exchange is fundamental to maintaining the mutual association between legumes and rhizobia. Rhizobia provide fixed N to their host plants, while legumes supply carbohydrates and all essential mineral nutrients to the rhizobia (Clarke et al. 2014; Streeter 1991; Udvardi and Poole 2013). This nutrient exchange primarily occurs within the root nodules and is facilitated through two pathways: the symplastic and apoplastic routes. In the symplastic pathway, nutrients are transported directly between cells via plasmodesmata, enabling efficient intracellular movement (Cao et al. 2022). By contrast, the apoplastic pathway involves nutrient diffusion through the extracellular space. When the apoplastic pathway is blocked, such as by the Casparian strip in the endodermis, nutrients are redirected into the symplastic pathway. These two pathways work synergistically to regulate nutrient exchange between the nodules and the host plant (Geldner 2013; Li et al. 2024; Roberts and Oparka 2003).

Nodules consist of various cell types, namely the epidermis, outer cortex cells, inner cortex cells, vascular bundles, infected cells (ICs), and uninfected cells (UCs). Each cell type plays a distinct role in N fixation by nodules. Nutrients in nodules are derived from direct uptake through the epidermis and outer cortex cells from the soil, as well as from transport via the plant vascular system. Ultimately, these nutrients are delivered to the ICs, where they are used for N fixation (Li et al. 2024). ICs are the primary site of N fixation and contain the oxygen-binding protein leghemoglobin, which lowers the oxygen concentration within nodules and thus protects nitrogenase from oxygen damage (Liu et al. 2023). Although UCs are rarely studied, they are known to contribute to the conversion of ammonium into organic N (Ye et al. 2022) and participate in iron (Fe) storage, providing essential support for SNF (Zhou et al. 2024).

Notably, inorganic N sources in the soil can inhibit nodulation and N fixation activity. Legumes employ a regulatory mechanism known as the autoregulation of nodulation (AON) that fine-tunes the extent of nodulation in response to external N availability, preventing excessive carbon consumption under N-sufficient conditions (Qiao et al. 2023; Streeter and Wong 2008). High levels of inorganic N can induce the senescence of mature nodules, which stops nutrient transfer from the host plant to nodules (Van de Velde et al. 2006). Although the precise mechanisms underlying N-induced suppression of SNF remain incompletely understood, potential factors include disruption of nutrient transport, interference with nodule development, and modulation of plant hormone concentrations (Zhou et al. 2024).

In this study, we used a symplastic tracing approach and conducted ultrastructural observations to elucidate the role of UCs in nutrient storage and transport within root nodules. We discovered an extensive network of plasmodesmata connecting ICs and UCs that dynamically regulates nutrient allocation. Nutrients transported via the vascular system are initially stored in UCs and then distributed to ICs based on their demand, ensuring precise control of N fixation. High N levels lead to callose deposition at plasmodesmata, impeding nutrient transport and diminishing N fixation efficiency. These findings provide new insights into the regulatory mechanisms of SNF and highlight the importance of UCs in optimizing SNF.

## Results

### Uninfected cells play a role in nutrient storage

**Fig. 1:**
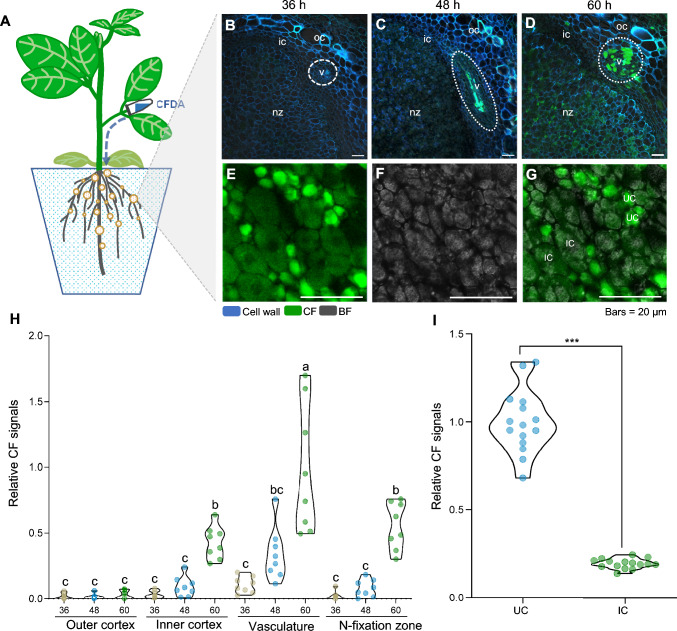
Assessing transport routes in soybean nodules with carboxyfluorescein diacetate (CFDA). **A** Diagram illustrating the principle underlying the CFDA symplastic loading test. Soybean roots are inoculated with rhizobia and cultured in a low-N (0.5 mM N) nutrient solution for 21 days. A section of the oldest trifoliolate leaf is removed with a razor, and its petiole is infiltrated with a CFDA solution. Nodules are collected 36 h, 48 h, and 60 h later for sectioning and examination of carboxyfluorescein (CF) signal. **B–G** CF signal distribution in nodules. **B–D** Cross sections of wild-type nodules at 36 h (**B**), 48 h (**C**), or 60 h (**D**) after infiltration with CFDA. oc, outer cortex; ic, inner cortex; v, vasculature (encircled by dotted lines); nz, N-fixation zone. **E–G** Magnified views of the N-fixation zone in nodules at 60 h after infiltration with CFDA. Green, CF signals; cyan, cell walls stained with calcofluor white; gray, bright field (BF). IC, infected cell; UC, uninfected cell. **H** Quantification of relative CF signals in outer cortex cells, inner cortex cells, vasculature cells, and the N-fixation zone. *n* = 8. Vasculature cells CF signals of 60 h was used as an internal standard. **I** Quantification of relative CF signals in ICs and UCs. *n* = 15. Different letters in **(H)** indicate significant differences (*P* < 0.05) in multiple comparisons tests by one-way ANOVA. Asterisks in **(I)** indicate significant differences compared to UCs (***P ≤ 0.001; two-sided *t*-test)

To simulate nutrient transport from the host plant to rhizobia, we employed the symplastic movement tracer carboxyfluorescein diacetate (CFDA) to observe and model the transport and storage status of nutrients within nodules (Knoblauch et al. 2015). Specifically, we introduced CFDA by injecting a CFDA solution into the petiole of the oldest trifoliate leaf, where it is rapidly cleaved by intracellular esterases to produce the fluorescent, membrane-impermeable compound carboxyfluorescein (CF). CF can therefore only diffuse and reach the nodules through the symplastic pathway (Fig. 1A). We used the soybean cultivar ‘Williams 82’, the roots of which had been inoculated with rhizobia for 21 days before assessing CF accumulation in its nodules (Fig. S1A, B). We excised a section of the oldest trifoliolate leaf with a razor and infiltrated the petiole with a CFDA solution (Fig. S1C). We waited 36–60 hours before collecting stem samples, leaf samples, and root nodules, to assess the presence and intensity of the CF signal. We observed that CF can quickly move to the phloem in the stem and leaves (Fig. S1D–K), although we detected no significant CF signal in nodules at 36 hours after infiltration. However, at 48 hours, a distinct CF signal began to appear in the vascular bundles of nodules. This signal strength intensified at 60 hours, with the inner cortex and N-fixation zone also displaying prominent CF signals (Fig. 1B–H). Notably, the intensity of the CF signal was significantly higher in the UCs of the N-fixation zone than in the ICs (Fig. 1G, I), suggesting that once CF enters the N-fixation zone, it predominantly accumulates in UCs. These results suggest that UCs may serve as a temporary storage site for nutrients transported from the host plant before their final delivery to ICs.

### Plasmodesmata exist between ICs and UCs but are absent between ICs

**Fig. 2:**
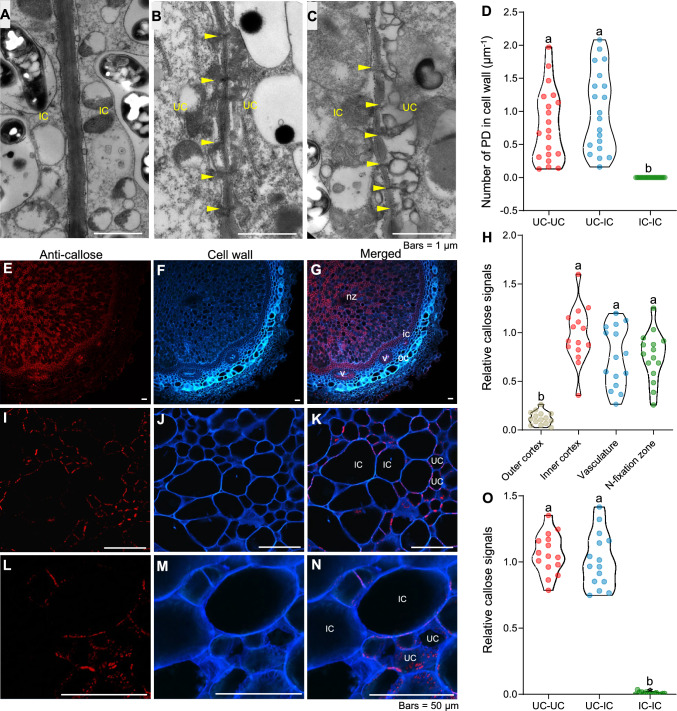
Distribution of plasmodesmata in soybean nodules.** A–C** Transmission electron microscopy (TEM) observations of plasmodesmata between infected cells (ICs) and uninfected cells (UCs) in nodules. Yellow triangles in (**B**) and (**C**) indicates plasmodesmata. **D** Number of plasmodesmata (PD) in the cell wall, calculated from TEM images. *n* = 20, for each type of cell. **E–G**, Callose deposition in the N-fixation zone and is magnified areas (**I–N**). Red, signal from the anti-callose antibody; cyan, cell walls stained with calcofluor white; oc, outer cortex; ic, inner cortex; v, vasculature; nz, N-fixation zone. **H** Quantification of relative callose signals in outer cortex cells, inner cortex cells, the vasculature, and the N-fixation zone. *n* = 15, individually differnet nodules Callose signals of inner cortex cells were used as an internal standard. **O** Quantification of relative callose signals between UCs and UCs (UC–UC), UCs and ICs (UC–IC), and ICs and ICs (IC–IC). *n* = 15, from different cells. The roots of soybean seedlings were inoculated with rhizobia and cultured in a low-N (0.5 mM N) nutrient solution for 21 days. Different letters in (**D**, **H**, **O**) indicate significant differences (*P* < 0.05) in multiple comparisons tests by one-way ANOVA

Since CF primarily diffuses into nodules through plasmodesmata, we hypothesized that there might be differences in the distribution of plasmodesmata among different cell types within nodules. To test this hypothesis, we examined the ultrastructure of soybean nodule cells using transmission electron microscopy (TEM). We observed a near-complete absence of plasmodesmata between ICs, while we counted abundant plasmodesmata between ICs and UCs, as well as between adjacent UCs (Fig. 2A–D). As previous studies have shown that callose deposition occurs at plasmodesmata (Cao et al. 2022; Wu et al. 2018), we used immunostaining with callose-specific antibodies to investigate callose distribution. We detected significant differences in callose deposition across different nodule regions and cell types. Specifically, callose deposition was prominent in the vascular bundles, endodermis, and N-fixation zone of nodules, but absent in the outer cortex (Fig. 2E–H). Furthermore, within the N-fixation zone, we observed no callose deposition between adjacent ICs, whereas we detected callose deposition between ICs and UCs, as well as between adjacent UCs (Fig. 2I–O). These findings align with the TEM observations above, suggesting that ICs cannot communicate or exchange nutrients directly with each other. Instead, they likely interact only with adjacent UCs for information and nutrient exchange.

### The permeability of plasmodesmata influences the mineral nutrient import into infected cells

**Fig. 3:**
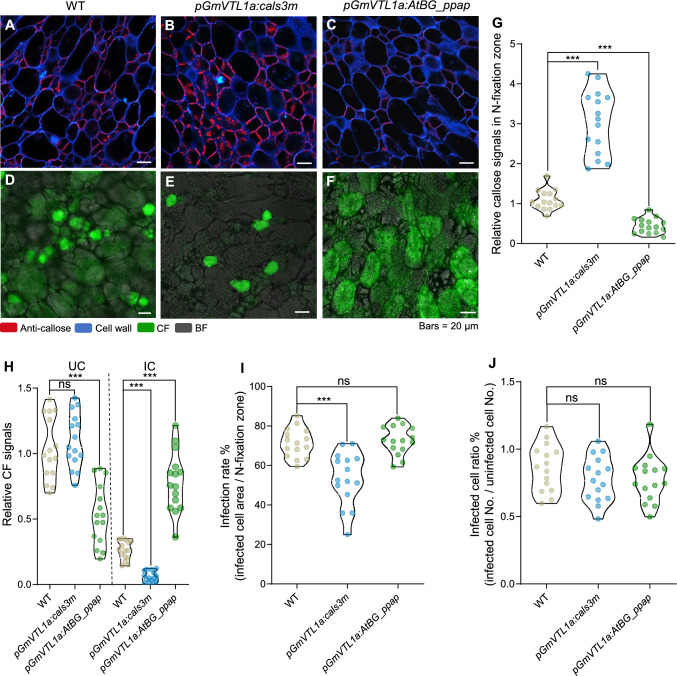
The permeability of plasmodesmata regulates carboxyfluorescein (CF) transport and infected cell development.** A–F** Callose deposition (**A–C**) and CF signal distribution (**D–F**) in transgenic nodules. Composite wild-type (WT), *proGmVTL1a:cals3m*, and *proGmVTL1a:AtBG_ppap* transgenic plants at 21 days post inoculation (dpi) were infiltrated with carboxyfluorescein diacetate (CFDA) for 60 h. Red, signals from the anti-callose antibody; cyan, cell walls stained with calcofluor white; green, CF signals; gray, bright field (BF). **G** Quantification of relative callose signals in the N-fixation zone. *n* = 15. **H** Quantification of relative CF signals in uninfected cells (UCs) and infected cells (ICs). *n* = 15. **I** Infection rate, as indicated by IC area within the N-fixation zone. *n* = 15. **J** Ratio of IC number to UC number. *n* = 15. Asterisks in (**G–J**) indicate significant differences compared to WT (***P ≤ 0.001; two-sided *t*-test); ns, not significant

To further investigate the regulatory role of plasmodesmata in mineral nutrient transport to ICs, we used the promoter of *VACUOLAR IRON TRANSPORTER-LIKE 1a* (*GmVTL1a*) (Liu et al. 2020), which is specifically expressed in ICs, to drive the expression of the callose synthase gene *cals3m* (a mutant of *CALS3* with gain-of-function missense mutations R84K and R1926K that overcome intrinsic negative regulation and additively enhance callose biosynthesis) (Vatén et al. 2011) or the gene encoding the callose-degrading enzyme AtBG_ppap (a putative beta-1,3-glucanase) from *Arabidopsis thaliana* (Benitez-Alfonso et al. 2013; Levy et al. 2007). We chose this approach to artificially modulate callose levels and thus regulate plasmodesmata permeability. Reverse-transcription quantitative PCR (RT-qPCR) analysis revealed high expression levels of *cals3m* and *AtBG_ppap* in the nodules derived from their respective transgenic hairy roots carrying *proGmVTL1a:cals3m* or *proGmVTL1a:AtBG_ppap* (Fig. S2A, B). When callose synthase abundance was artificially increased in *proGmVTL1a:cals3m* hairy roots, callose deposition in the N-fixation zone of nodules significantly increased compared to that in wild-type nodules (Fig. 3A, B). We also observed a block in CF transport in the nodules from *proGmVTL1a:cals3m* transgenic hairy roots, leading to a low CF signal within ICs (Fig. 3A, B, D, E, G). Conversely, when the levels of the callose-degrading enzyme were artificially induced, callose deposition in the N-fixation zone was significantly lower than that in wild-type nodules, alleviating any obstruction to CF transport. As a result, more CF moved into ICs, enhancing the CF signal in these cells (Fig. 3C, F–H). Furthermore, the artificial induction of callose deposition at plasmodesmata led to smaller ICs in the N-fixation zone, although the number of ICs remained unchanged (Fig. 3I, J). This result suggests that the obstruction of mineral nutrient transport through plasmodesmata indirectly affects the development of ICs.

### Plasmodesmata permeability affects mineral nutrient homeostasis and the SNF ability of nodules

**Fig. 4:**
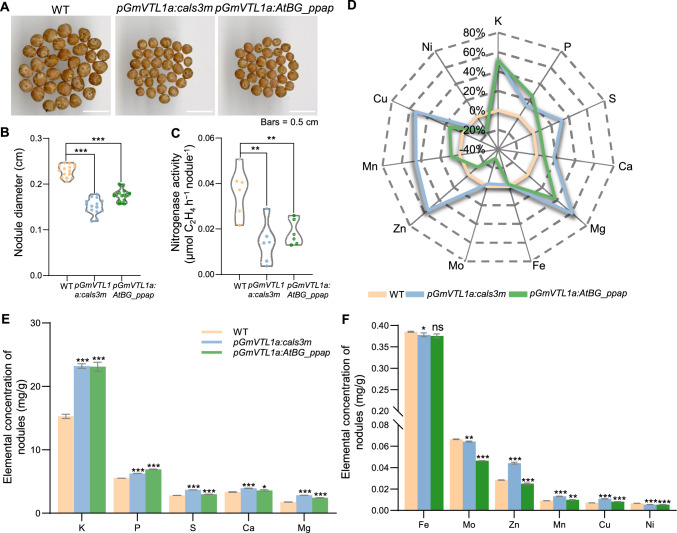
The permeability of plasmodesmata affects mineral homeostasis and the symbiotic nitrogen fixation ability of nodules. **A** Representative photographs of nodules from composite wild-type (WT), *proGmVTL1a:cals3m*, and *proGmVTL1a:AtBG_ppap* transgenic plants. **B** Nodule diameter. *n* = 15. **C** Nitrogenase activity. *n* = 6. Nodules were collected at 21 days post inoculation (dpi) from composite WT, *proGmVTL1a:cals3m*, and *proGmVTL1a:AtBG_ppap* transgenic hairy roots for the assay. **D** Mineral content analysis of nodules, shown as a percentage difference from WT (100 × [element conc. in transgenic hairy root − element conc. in WT] / element conc. in WT). *n* = 3. **E**, **F** Contents of macronutrients (**E**) and micronutrients (**F**) in nodules. *n* = 3, independent pools, 1g of nodules per pool. Asterisks in (**B**, **C**, **E**, **F**) indicate significant differences compared to WT (*P ≤ 0.05; **P ≤ 0.01; ***P ≤ 0.001; two-sided *t*-test). ns, not significant

We next examined the nodules and established that modulating plasmodesmata permeability by altering callose levels in either direction resulted in smaller nodules (Fig. 4A, B), with lower nitrogenase activity (Fig. 4C). We then explored the mineral contents of all nodules using inductively coupled plasma mass spectrometry (ICP–MS). We observed that altering callose levels significantly disrupted the mineral content of nodules (Fig. 4D). Specifically, the concentrations of the macronutrients potassium (K), phosphorus (P), sulfur (S), calcium (Ca), and magnesium (Mg) were significantly higher than those in wild-type nodules (Fig. 4E). Among the micronutrients, molybdenum (Mo) and nickel (Ni) concentrations were lower than those in wild-type nodules, while those of manganese (Mn) and copper (Cu) were slightly higher in the nodules harbored by transgenic hairy roots. Zinc (Zn) levels were higher in nodules with greater callose deposition and lower in those with lower callose amounts (Fig. 4F). These findings indicate that the permeability of plasmodesmata between ICs and UCs regulates mineral nutrient homeostasis and overall nodule development.

### High N inhibits nutrient exchange by lowering plasmodesmata permeability

**Fig. 5:**
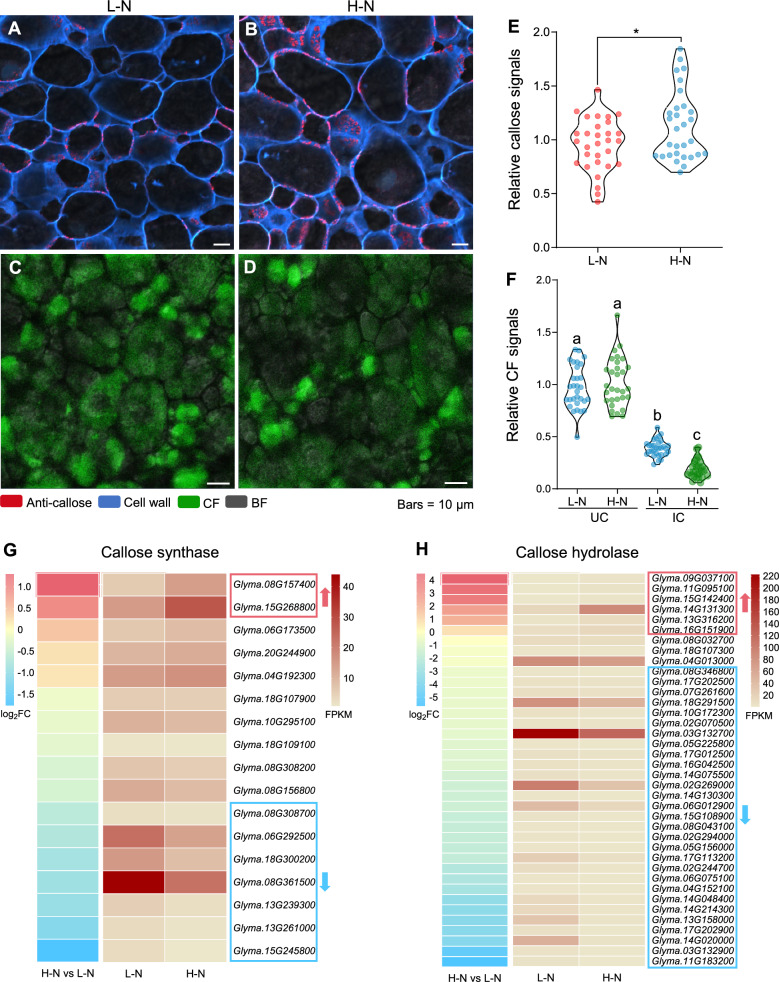
High N results in lower plasmodesmata permeability and impairs carboxyfluorescein (CF) transport. **A–D** Callose deposition and CF signal distribution in the N-fixation zone. Wild-type soybean seedlings were inoculated with rhizobia, cultured in a low-N (L-N, 0.5 mM N) nutrient solution for 18 days, and then cultured in a high-N (H-N, 20.5 mM N) nutrient solution for 3 days. **A**, **B** Callose deposition; **C**, **D** CF signal distribution 60 h after carboxyfluorescein diacetate (CFDA) injection. Red, signals from the anti-callose antibody; cyan, cell walls stained with calcofluor white; green, CF signals; gray, bright field (BF). **E** Quantification of relative callose signals in the N-fixation zone. *n* = 30. **F** Quantification of relative CF signals in uninfected cells (UCs) and infected cells (ICs). *n* = 30. **G**, **H** Heatmap representation of expression levels for genes related to callose biosynthesis (**G**) or degradation (**H**) in soybean nodules. Fragments Per Kilobase of transcript per Million mapped reads (FPKM) values are shown. Up or down arrows indicate genes that are upregulated or downregulated by more than 1.5-fold, respectively. Asterisks in (**E**) indicate significant differences compared to L–N (*P < 0.05; two-sided *t*-test). Different letters in (**F**) indicate significant differences (*P* < 0.05) in multiple comparisons tests of one-way ANOVA

Considering that high N levels can hinder nutrient transport in nodules, we wondered whether plasmodesmata permeability would be affected by high-N conditions, potentially contributing to this inhibition. To test this idea, we examined callose deposition and CF transport in the N-fixation zone of wild-type nodules under high-N conditions. Our observations revealed greater callose deposition in the N-fixation zone and a lower CF signal in ICs after high N supply (Fig. 5A–F), suggesting that high N may impede nutrient exchange by diminishing plasmodesmata permeability.

Furthermore, a transcriptome deep-sequencing (RNA-seq) analysis of public data from nodules exposed to low-N or high-N conditions (Zhou et al. 2024) identified changes in the expression of genes related to callose metabolism. Specifically, two callose synthase–related genes were upregulated by high N, while another seven were downregulated under the same conditions (Fig. 5G). Similarly, six callose degradation–related genes were upregulated, while another 29 were downregulated (Fig. 5H). These findings suggest that high-N stress may regulate plasmodesmata permeability by modulating the expression of genes involved in callose biosynthesis and degradation, thereby affecting nutrient exchange.

## Discussion

During SNF, ICs are the primary sites of rhizobium colonization and N fixation, and have thus garnered much attention from scientists. By contrast, the functions of their neighboring UCs have often been overlooked. Structurally, each IC is connected to at least one UC, with UCs accounting for about one-fifth of the total N-fixation zone area (Selker and Newcomb 1985). These UCs are not merely cells that have yet to be colonized by rhizobia; rather, they are specifically differentiated tissues with functions distinct from those of ICs. The relationship between ICs and UCs is somewhat analogous to that between sieve tube elements and companion cells in the phloem. Before our study, Brown et al. (1995) proposed that UCs formed ray-like structures potentially facilitating nutrient transfer to ICs, suggesting UC–IC connectivity. However, the composition and precise localization of these ray-like structures were not clearly presented. Our ultrastructural analysis revealed a complete absence of plasmodesmata between ICs (Fig. 2A), contrasting with the abundant plasmodesmata at UC–IC and UC–UC interfaces (Fig. 2B–C). Transgenic modulation of callose deposition independently demonstrated the critical role of this polymer in regulating plasmodesmata-mediated nutrient exchange in nodules. Schubert et al. (2010) reported the existence of inter-IC plasmodesmata in indeterminate nodules of Durango root (*Datisca glomerata*). Unlike the determinate nodules of soybean, indeterminate nodules simultaneously contain early immature, mature, and senescent zones, making it difficult to ascertain whether these two types of nodules share identical symplastic pathways. This structural difference likely leads to distinct nutrient exchange mechanisms between these two types of nodules.

Recent studies utilizing single-cell RNA sequencing have revealed that asparagine, a key compound for N storage and transport in plants, is primarily synthesized in UCs. This finding suggests that UCs may play a critical role in the N assimilation pathway within nodules (Ye et al. 2022). Additionally, our previous research identified a pair of putative Fe transporters, Natural Resistance-Associated Macrophage Protein 2a (GmNRAMP2a) and GmNRAMP2b, that are specifically localized to the vacuolar membrane of UCs. These proteins are responsible for supplying Fe to ICs when needed (Zhou et al. 2024), supporting a role for UCs in Fe homeostasis within nodules. In this study, we observed that the symplastic tracer CF, transported from the shoot, predominantly accumulates in UCs (Fig. 1G). Furthermore, plasmodesmata are absent between ICs, suggesting that their only means of communication is through adjacent UCs. These results suggest that Fe, and likely all nutrients that are delivered from the host plant via the symplastic pathway, may first be stored in UCs before being allocated to neighboring ICs as required. These findings highlight the additional role of UCs in nutrient storage and distribution in root nodules.

Plants can regulate transport through plasmodesmata by modulating the biosynthesis and degradation of callose, thereby altering intercellular permeability to control biochemical signaling and nutrient transport in response to environmental stimuli (Faulkner 2018; O’Lexy et al. 2018). In this study, ultrastructural observations revealed significant differences in the distribution of plasmodesmata among different cell types within nodules. Notably, plasmodesmata were absent between ICs, while they were abundant between UCs and ICs. This feature may provide a more efficient channel for nutrient exchange between the host plant and rhizobia. Furthermore, we artificially induced callose deposition at the plasmodesmata between ICs and UCs, which validated the role of plasmodesmata permeability in nutrient transport into ICs. Indeed, we determined that altering callose levels substantially disrupted mineral homeostasis (Fig. 4D). In particular, Mo and Ni concentrations were lower than those in wild-type nodules (Fig. 4F). Mo is an essential component of molybdoenzymes such as nitrogenase, xanthine dehydrogenase, and nitrate reductase (Kaiser et al. 2005). Ni acts as an activator of the enzyme urease, thereby facilitating the hydrolysis of urea into NH_3_ (Fabiano et al. 2015). Therefore, the lower Mo and Ni concentrations observed in nodules with altered callose deposition may directly affect nitrogenase activity, thereby influencing the efficiency of SNF in soybean nodules. Additionally, callose deposition was higher in the N-fixation zone of nodules exposed to high-N conditions, which was accompanied by a drop in CF signal in ICs (Fig. 5B, D). This finding suggests that high-N stress may lower plasmodesmata permeability by enhancing callose deposition, thereby impairing nutrient exchange and transport. Together, these findings highlight the critical role of plasmodesmata in mediating nutrient dynamics and their regulation by environmental stress.

Callose-mediated regulation of plasmodesmata permeability may play important roles in modulating mineral nutrient exchange between ICs and UCs in root nodules, and it potentially contributes to the suppression of SNF under high-N conditions. While our findings demonstrate a clear correlation between callose deposition and nutrient transport, the precise molecular mechanisms underlying this regulation remain to be elucidated. An analysis of single-cell RNA-seq data from soybean root nodules (Liu et al. 2023) revealed distinct gene expression patterns between ICs and UCs. While 311 genes were upregulated in ICs (including the callose synthase gene Glyma*.*08G156800), UCs showed an upregulation of 966 genes, including three callose hydrolase genes (Glyma*.*14G131300, Glyma.14G214300, Glyma.02G269000) and one callose synthase gene (Glyma.18G300200). Whether these genes directly mediate the observed changes in plasmodesmata permeability requires further investigation.

In conclusion, our study reveals a critical role for UCs in the storage and release of nutrients within nodules and elucidates the importance of plasmodesmata between UCs and ICs in nutrient transport. These findings suggest that UCs may serve as a central hub for regulating N fixation, receiving signals and responding accordingly to precisely control the N fixation efficiency and productivity of ICs.

## Materials and Methods

### Plant materials and growth conditions

The soybean (*Glycine max*) cultivar ‘Williams 82’ was used in this study. Seeds were surface sterilized by exposure to chlorine gas overnight and subsequently germinated in vermiculite. Four-day-old soybean seedlings were used in the experiments. The nutrient solution for soybean growth was based on Peng et al. (2018). The standard nutrient solution contained 500 μM KH_2_PO_4_, 500 μM MgSO_4_·7H_2_O, 500 μM (NH_4_)_2_SO_4_, 1900 μM KNO_3_, 1200 μM Ca(NO_3_)_2_·4H_2_O, 40 μM EDTA-Fe, 0.5 μM CuSO_4_·5H_2_O, 1 μM MnSO_4_·H_2_O, 1 μM ZnSO_4_·7H_2_O, 1 μM (NH_4_)_6_Mo_7_O_24_·4H_2_O, 3 μM H_3_BO_3_. Seedlings were cultivated in a growth chamber under a 12-h light/12-h dark photoperiod and a 30 °C/25 °C (day/night) cycle, and maintained at a humidity level of 60%. The nutrient solution was renewed every 2 days, and the pH was adjusted to 5.8. Soybean roots were continuously aerated using an air pump throughout the experiment.

To artificially induce callose biosynthesis and degradation in the cells of root nodules, the vector pFGC5941-35s:GFP was modified to generate a *proGmVTL1a:GFP* construct by replacing the cauliflower mosaic virus (CaMV) 35S promoter with a 2,490-bp fragment of the *GmVTL1a* (Glyma.05G121600) promoter using *Mlu*I and *Asc*I. The *GmVTL1a* promoter was amplified by PCR using specific primers (Table S1). The resulting modified plasmid was then used as a backbone to replace the *GFP* open reading frame with *cals3m* or *AtBG_ppap* using *Asc*I and *Sma*I to obtain the *proGmVTL1a:cals3m* and *proGmVTL1a:AtBG_ppap* constructs, respectively. The *cals3m* fragment from a pDONR221*-cals3m* vector (Curtis and Grossniklaus 2003). The full-length coding sequence of *AtBG_ppap* (At5g42100) was amplified by PCR using specific primers (Table S1).

### Rhizobia inoculation

The rhizobial strain *Sinorhizobium fredii* CCBAU 45436 was cultured on TY medium (0.5% [w/v] tryptone, 0.3% [w/v] yeast extract, 0.06% [w/v] CaCl_2_) solidified with 1.5% (w/v) agar at 28 °C for 2 days. Fresh rhizobia were scraped off using a spreader, placed into liquid TY medium, and cultured at 28 °C with shaking at 220 rpm until the OD_600_ reached 1. The bacterial suspension was then centrifuged at 7,000 rpm for 5 min at room temperature to collect the bacteria. The bacterial pellet was resuspended in deionized water to an OD_600_ of 0.5. The resuspended bacterial solution was mixed with sterile vermiculite to inoculate the soybean seedlings. After 21 days of co-cultivation in a low-N nutrient solution (1/10 N strength of the standard nutrient solution), the root nodules were harvested.

### Hairy root transformation

To generate transgenic soybean composite plants (i.e., wild-type shoots with transgenic roots), the hypocotyl injection method was employed for hairy root transformation, with modifications based on the protocol described by Guo et al. (2011). Specifically, the hypocotyls of 4-day-old soybean seedlings with unfolded cotyledons were inoculated with *Agrobacterium rhizogenes* strain K599 harboring the desired construct. The inoculated seedlings were then maintained in a high-humidity environment until the emerging hairy roots reached a length of approximately 5 cm. True transgenic hairy roots were identified through GUS staining for the experiments.

### CFDA transport and callose assay

Carboxyfluorescein diacetate (CFDA) is a commonly used symplastic tracing agent that produces the fluorescent and membrane-impermeable compound carboxyfluorescein (CF) after cleavage by intracellular esterases (Knoblauch et al. 2015). To simulate nutrient transport from the host plant to rhizobia, CFDA was used to observe and model the transport and storage status of nutrients within the nodules. Hairy roots were inoculated with rhizobia and cultured in a low-N (0.5 mM N) nutrient solution for 21 days, followed by culture in a high-N (20.5 mM N) nutrient solution for 3 days. Subsequently, one of the oldest trifoliolate leaves was excised using a razor blade, and its petiole was infiltrated with 200 mM CFDA (Solarbio). Nodules were collected at 36 h, 48 h, and 60 h after CFDA infiltration and embedded in 5% (w/v) agar for sectioning into 50-μm thick slices using a microtome (Leica RM2235; Leica Microsystems GmbH, Wetzlar, Germany) at 4°C. CF signals were visualized and imaged using a confocal microscope (LSM880; Carl Zeiss), with a 488-nm argon laser for excitation and a 535 ± 20 nm filter for emission detection. For callose assays, nodule samples were fixed and prepared for immunostaining according to previously published methods (Liu et al. 2020). Briefly, nodule samples were fixed in a solution of 4% (w/v) paraformaldehyde and 60 mM sucrose, buffered with 50 mM cacodylic acid at pH 7.4 for 2 hours. After three-time washing with phosphate-buffered saline (PBS; consisting of 10 mM Na_2_HPO_4_, pH 7.4, 138 mM NaCl, and 2.7 mM KCl), the samples were embedded in 5% (w/v) agar and sectioned into 50-µm-thick slices. The sections were then treated with PBS containing 0.3% (v/v) Triton X-100 for 2 h. After three-time washing, the sections were incubated overnight in PBS with 5% (w/v) bovine serum albumin (BSA) and the primary antibody. Subsequently, the sections were three-time washed with PBS before being incubated with secondary antibody for 2 h. Afterward, the samples were four-time washed with PBS and mounted with 50% (v/v) glycerol in PBS. All immunostaining procedures were conducted at room temperature. Anti-callose antibodies (1:100; 1,3-β-glucan; Biosupplies) and Alexa Fluor 555 goat anti-mouse IgG (1:3000; Molecular Probes) were used as primary and secondary antibodies, respectively. The CF and callose fluorescence signals in different tissues were quantified using ImageJ software (Version 1.53t, Computer software, 2020, National Institutes of Health).

### Transmission electron microscopy observations

Nodule ultrastructure was observed according to published methods (Liu et al. 2020). Briefly, nodule samples were first fixed in 2.5% (v/v) glutaraldehyde and 1% (w/v) osmium tetroxide. Fixed samples were dehydrated in a graded series of ethanol (50-100%). Samples were then embedded in Spurr’s resin and sliced into 70 nm thick sections before being photographed in transmission electron microscopy**.**

### Determination of nitrogenase activity in nodules

The nitrogenase activity of nodules was assessed using acetylene reduction assays, as described by Siczek and Lipiec (2011). Specifically, two or three large nodules were excised from a single plant root and placed in a 10-mL bottle sealed with Parafilm. Then, 1/10 (v/v) of the gas from the bottles was replaced with acetylene using a syringe. After incubating the samples at room temperature for 2 h, 1 mL of 0.5 M NaOH was injected into the bottle to terminate the reaction. A 0.3-mL gas sample from each bottle was taken with a syringe and used for ethylene determination using a gas chromatograph (GC-2014, SHIMADZU, Kyoto, Japan). The nitrogenase activity was calculated by the equation below: The nitrogenase activity (µmol C_2_H_4_ g^-1^ h^-1^) = C (C_2_H_4_, g l ^-1^) × V (l)/ (Mw (28.06) × nodule FW (g) × T (h)) × 10^6^.

### Quantitative gene expression analysis

Nodules at 21 days post inoculation (dpi) were collected for total RNA extraction using a TransZol Up Plus RNA Kit (TransGen Biotech). After extraction, first-strand cDNA synthesis was performed using ReverTra Ace qPCR RT Master Mix (TOYOBO), which includes a genomic DNA (gDNA) remover, in accordance with the manufacturer's instructions. Subsequently, gene expression levels were analyzed using quantitative PCR (qPCR) with TransStart Top Green qPCR SuperMix (TransGen Biotech). The housekeeping gene *EF-1a* was used as an internal control. Expression levels were quantified and normalized using the 2^−ΔΔCt^ method. Primers used for RT-qPCR are listed in Table S1.

### Statistical analysis

Experimental data were processed and analyzed using Microsoft Excel 2019 and GraphPad Prism 8.3 to calculate means, standard deviations, and generate graphical representations. Statistical analyses were conducted using SPSS Statistics 19.0 (IBM, Armonk, NY, USA), employing one-way analysis of variance (ANOVA) followed by Tukey’s test, or unpaired two-tailed *t*-tests as appropriate.

## Supplementary Information

Below is the link to the electronic supplementary material.

Supplementary file1 (DOCX 4167 KB)

Supplementary file2 (DOCX 18 KB)

## Data Availability

All data supporting the findings of this study are available within the article and supplementary files.
