# Supplementary material for: Nutrient storage and release in uninfected cells of soybean nodules support symbiotic nitrogen fixation in infected cells
Source: aBIOTECH. 2025 Sep 16;6(4):790–802. doi: 10.1007/s42994-025-00247-y (PMC12647401; doi:10.1007/s42994-025-00247-y)
Supplement: Supplementary file 1 — Supplementary file1 (DOCX 4167 KB) [file 42994_2025_247_MOESM1_ESM.docx]

**Supplementary Figure**


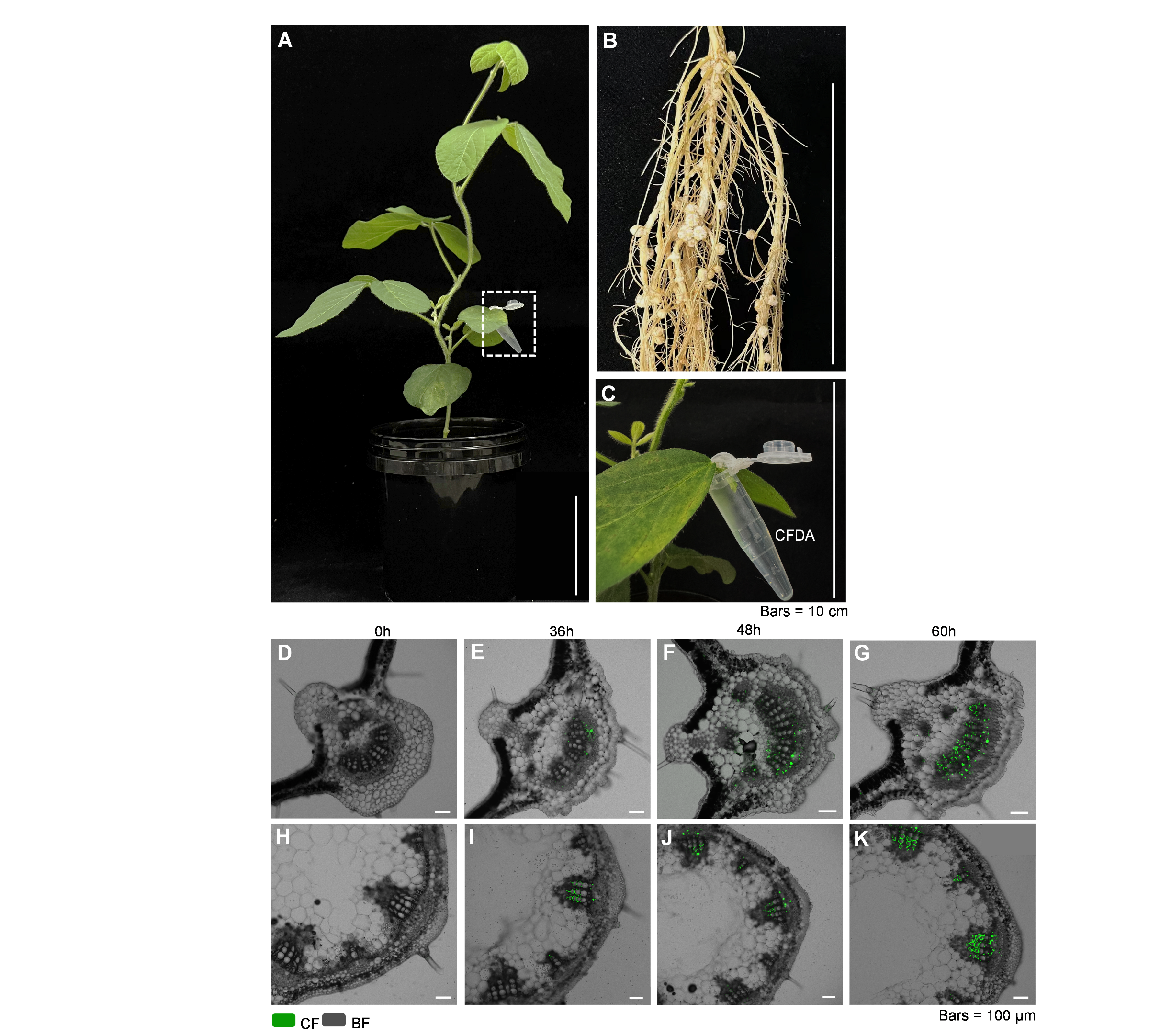


**Fig. S1** CF transport pathway in soybean shoots. **A-C** The profile of CFDA loading. One-week-old soybean seedlings were inoculated with rhizobia and cultured in a low-N (0.5 mM N) nutrient solution for 21 days. The status of shoots (**A**) and nodules (**B**) were shown. A section of the oldest trifoliolate leaf was removed with a razor, and its petiole was infiltrated with CFDA solution (**C**). **D-G** CF signal distribution in leaf veins. **H-K** CF signal distribution in stems. Soybean was infiltrated with CFDA solution for 0, 36, 48, and 60 hours, and then the stems and the second trifoliolate leaves were sampled for observation. Green shows CF signals, gray areas show bright field.


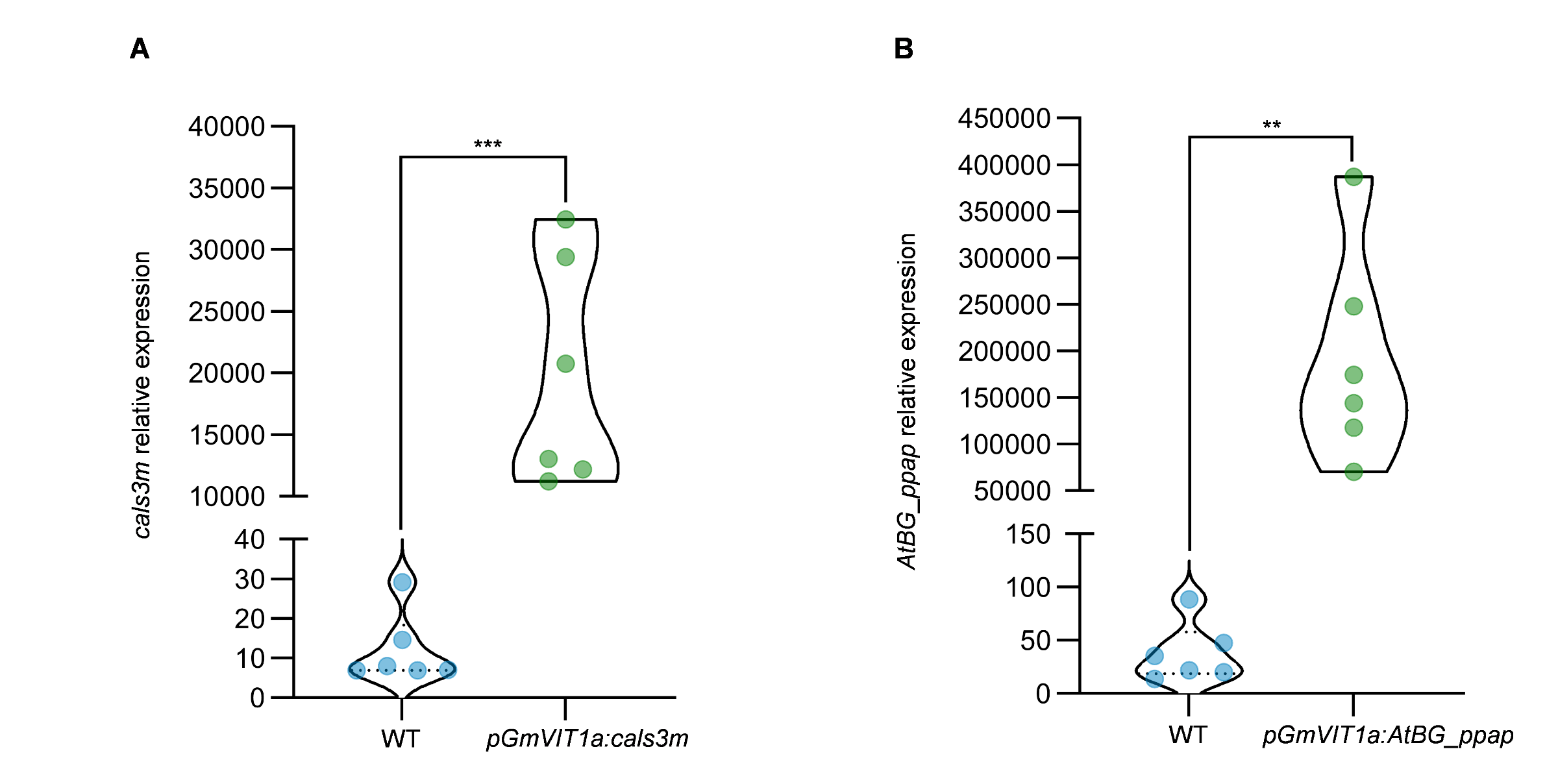


**Fig. S2** Target gene expression in transgenic nodules. **A** The gene expression levels of the *cals3m* in the nodules of WT and *pGmVIT1a:cals3m* transgenic lines. **B** The gene expression levels of the *AtBG_ppap* in the nodules of WT and *pGmVIT1a:AtBG_ppap* transgenic lines. Nodules at 21 dpi from WT, *pGmVTL1a:cals3m* and *pGmVTL1a:AtBG_ppap* transgenic lines were sampled for analysis. All the transgenic lines used are hairy root transformants of soybean. Relative gene expression levels in (**A-B**) were determined by real-time RT-PCR. *EF-1a* was used as an internal standard. n = 6 biologically independent replicates. Asterisks in (**A-B**) indicate significant differences compared with WT (***P* ≤ 0.01; ****P* ≤ 0.001; two-sided *t*-test).
