# Supplementary material for: Nutrient storage and release in uninfected cells of soybean nodules support symbiotic nitrogen fixation in infected cells
Source: aBIOTECH. 2025 Sep 16;6(4):790–802. doi: 10.1007/s42994-025-00247-y (PMC12647401; doi:10.1007/s42994-025-00247-y)
Supplement: Supplementary file 2 — Supplementary file2 (DOCX 18 KB) [file 42994_2025_247_MOESM2_ESM.docx]

**Table S1 Primers used in this study**

| Primer names and usage | | Primer sequences (5'-3') |
| --- | --- | --- |
| Primers for pGmVTL1a: GFP vector construction | pGmVTL1a: GFP-F | GCAAGAGCAGCAGCTGACGCGTTTGATTCTTCGCGTG |
|  | pGmVTL1a: GFP-R | GCCCTTGCTCACCATGGCGCGCCGTTTAGACGTTGT |
| Primers for pGmVTL1a: cals3m vector construction | pGmVTL1a: cals3m-F | ACAACGTCTAAACGGCGCGCC ATGTCTGCTACGAGA |
|  | pGmVTL1a: cals3m-R | ACTCTAGGGACTAGTCCCGGG TCATTCCTTGTTTCG |
| Primers for pGmVTL1a: AtBG_ppap vector construction | pGmVTL1a: AtBG_ppap-F | ACAACGTCTAAACGGCGCGCCATGGCTTCTTCTTCT |
|  | pGmVTL1a: AtBG_ppap-R | ACTCTAGGGACTAGTCCCGGGTTACAACCGAAGCTT |
| Primers for RT-qPCR | cals3m-RT-F | GGCGGATTATACGAACTCAGAC |
|  | cals3m-RT-R | GGGCAATCTCAACAAGAGATGA |
|  | AtBG_ppap-RT-F | CGTCTTTGCTCTCTTTAACGAG |
|  | AtBG_ppap-RT-R | GAAATGGACATGTAATCCGGTG |
